# Inhalation of Iodine in Asthma

**Published:** 1877-10

**Authors:** J. G. Westmoreland

**Affiliations:** Atlanta, Ga.


ATLANTA

MEDICAL AND SURGICAL JOURNAL

VOL. XV.] OCTOBER—1877. [NO. 7

### Original Communications.

### INHALATION OF IODINE IN ASTHMA.

BY J. G. WESTMORELAND, M.D., ATLANTA, GA.

The disease known by the name which heads this article has engaged the attention of pathologists for a long time, and still the exact condition of the organs concerned, is an unsettled question. The truth is, the prominent symptom giving origin to the name, is the result of various and very different pathological conditions. Difficult or laborious breathing of a particular kind is called asthmatic, while dyspnœa is the term applied to difficult or obstructed breathing from mechanical or other cause, not exhibiting the peculiarities of asthma. Literally, both terms alike denote imperfect respiration, and it is only by custom that they are made to designate different forms or varieties of laborious breathing. Asthmatic breathing is characterized by a wheezing sound, audible at a distance from the subject, bronchial mucous or soft sibilant rale, loose cough, with sometimes copious mucous expectoration. It is distinguished from dyspnœa caused by hydrothorax or other cause lessening the capacity of the chest, by the dry cough, dry, sibilant rale, and almost entire want of expectoration, in the latter.

The generally recognized condition in asthma is that of bronchial spasmodic contraction, but quite a diversity of opinion exists touching the direct cause of the contraction. One says it is the result of accidental irritating cause, operating on the bronchial mucous membrane, and extending to the muscular tissue, in a subject predisposed to spasm of the bronchial muscles; others think the moving cause exists in the nervous system, and that the derangement is first felt in the nervous centers; while others, again, conclude that reflex nervous impression on the bronchial muscles from local irritation in the mucous membrane is the only reasonable explanation.

Asthmatic trouble is always paroxysmal, and the attacks occur most frequently at night, leaving the patient, in many instances, comparatively free from suffering during the daytime. Some subjects of the disease have annual attacks, at a particular season, and exhibit decided evidences of constant irritation of the nasal mucous membrane. In such cases, coryza is the first symptom observed in the attack, and exists for several days before the paroxysms of difficult breathing appear. This form of the disease is known to the public by the name, "hay fever," or "hay asthma," when occurring at commencement of autumn, and "rose fever," when it comes on in the spring. Persons subject to this annual attack, particularly those in which it occurs about the last of summer, are generally free from all the symptoms of asthma during the whole year, except the month of September. In this form, local irritation, and in most instances, subacute inflammation of the respiratory mucous membrane exists throughout the bronchial and nasal passages, and yet, while the coryza is constantly present, difficult respiration occurs only occasionally, as in ordinary asthma.

Reasoning from analogy, we infer that we have a constant local irritation, or subacute inflammation, as the foundation of ALL forms of asthmatic spells. The opinion that the paroxysms of bad breathing are immediately produced by reflex nervous impression, in asthma of any kind, we think is fully sustained by fair and legitimate reasoning on the subject. The paroxysmal character of all reflex impressions leads us readily to suspect such influence to exist whenever periodicity is found in disease, without evidences of primary lesion of a nervous center. Particularly is this the case when irritation or inflammation is detected in a neighboring organ to that in which the paroxysmal manifestations are found. Sometimes the diseased organ exhibits reflex derangement, and even organs remote from the local lesion, have their functions disturbed in this way. Familiar examples are found in the development of tetanus by local irritation in the extremity of the body; hysterical pulmonary, cardiac, gastric, or uterine functional derangement, from uterine irritation; convulsions as the result of enteric trouble, etc. Bronchial irritation from exposure to cold in certain constitutions, may lead to functional disturbance of the respiratory tubes in the form of paroxysmal spasm, such as is found in a fit of asthma. Thus derangement of the respiratory organs is manifested in spasmodic contraction of the muscular coat of the bronchia from irritation of the mucous surface.

With this opinion of its pathology, we, some years ago, determined to test the effects of direct applications to the respiratory mucous membrane in asthma. Accordingly, about the first of September, for a case of "hay fever," the inhalation of iodine was advised. The subject, a lady of thirty-five years old and the mother of four children, had suffered regularly with the disease during the whole month of September, for a number of years. With the view of preventing the annual return she had taken daily about ten grains of quinine for a week. As usual, however, the symptoms were fully developed by the first, and the treatment was commenced about the fifth of September. One-fourth grain of morphine was given, and in an hour one grain of iodine was inhaled, by placing the remedy in a wide-mouthed small vial, sufficiently heated to change it into fumes, and inhaling through the nostrils. Notwithstanding the anodyne effect of the opiate, an unpleasant paroxysm of cough was produced, leaving the patient much relieved from the coryza and dyspnœa, however, during the succeeding twelve hours. The inhalation was repeated every twenty-four hours for several days, and afterwards less frequently for two weeks, with the effect of preventing any decided asthmatic paroxysms, during the night or day, as had been her habit.

Some bronchial and nasal trouble continued for three weeks, but such decided relief was afforded that the lady is delighted with the prospect of being rid, in future, of this distressing and unwelcome annual visitor.

More recently, a case of the same general character came under my observation, but had advanced so far towards the time of its spontaneous termination that no certain and decided results can be claimed. The inhalation of iodine was had daily, however, and followed by complete relief from the usual nightly paroxysms of asthma.

Patients suffering from this disease, and their physicians, generally feel disinclined to embarrass the already difficult breathing with the means proposed above, but my experience in the use of iodine inhalations, not only in asthma, but in the treatment of ordinary chronic bronchitis, catarrh, etc., justifies the opinion that no fears need be had of producing injurious effects; and by preparing the patient with an opiate an hour previous to the inhalation, the unpleasant cough attending it is measurably prevented.

This is the only local alterative or catheretic remedy that can be readily applied to the bronchial mucous membrane, and this the only form in which iodine can be inhaled in sufficient degree of concentration to relieve chronic disease permanently.

